# Traditional Chinese Medication Tongxinluo Attenuates Lipidosis in Ox-LDL-Stimulated Macrophages by Enhancing Beclin-1-Induced Autophagy

**DOI:** 10.3389/fphar.2021.673366

**Published:** 2021-06-25

**Authors:** Yifei Chen, Fangpu Yu, Yu Zhang, Mengmeng Li, Mingxue Di, Weijia Chen, Xiaolin Liu, Yun Zhang, Mei Zhang

**Affiliations:** ^1^ The Key Laboratory of Cardiovascular Remodeling and Function Research, Chinese Ministry of Education, Chinese National Health Commission and Chinese Academy of Medical Sciences, The State and Shandong Province Joint Key Laboratory of Translational Cardiovascular Medicine, Qilu Hospital of Shandong University, Jinan, China; ^2^ Department of Echocardiography, Beijing Anzhen Hospital, Capital Medical University, Beijing, China

**Keywords:** atherosclerosis, tongxinluo, macrophages, lipid metabolism, autophagy

## Abstract

Tongxinluo (TXL), a traditional Chinese medication, plays a key role in the formation and progression of plaques in atherosclerosis. The formation of foam cells by macrophages accelerates the destabilisation of plaques. In previous research, we had found that TXL significantly inhibits ox-LDL-induced apoptosis in macrophages *in vitro* by improving the dissociation of the Beclin-1-Bcl-2 complex. Therefore, here, we explored the effect of TXL on lipid metabolism in macrophages and the mechanism involved. To evaluate the role of TXL in atherosclerotic plaques, we construct the atherosclerotic animal model with lentiviral injection and performed immunofluorescence staining analysis *in vivo*. Western blot, immunofluorescence staining and microscopy were performed to elucidate the mechanism underlying TXL-mediated regulation of autophagy in THP-1 macrophages *in vitro*. Immunofluorescence assay revealed that TXL treatment inhibited lipid deposition in advanced atherosclerotic plaques. *In vitro* TXL treatment inhibited lipid deposition in THP-1 macrophages by enhancing autophagy via Beclin-1. TXL reversed the high expression of class I histone deacetylases (HDACs) induced by ox-LDL (*p* < 0.05). Compared with the TXL + ox-LDL group, TXL failed to promote intracellular lipid droplet decomposition after the addition of the histone deacetylase agonist. We found that TXL attenuates the accumulation of lipids in macrophage by enhancing Beclin-1-induced autophagy, and additionally, it inhibits the inhibitory effect of class I HDAC on the expression of Beclin-1.

## Introduction

Atherosclerosis is a chronic inflammatory disease caused because of lipid dysfunction that occurs in the walls of the large and middle arterial blood vessels (Lusis, 2000). In the initial stages of atherosclerosis, endothelial function is disturbed and apolipoprotein B lipoproteins, such as low-density lipoprotein (LDL), are retained in the subendothelium, while the endothelium is activated to secrete chemokines and monocyte adhesion molecules. After monocytes enter the vascular endothelium, they differentiate into macrophages, which take up the subcutaneous lipoproteins. With the accumulation of lipids in macrophages, large amounts of lipid droplets (LDs) accumulate in the cytoplasm of macrophages, which eventually transform into foam cells. As a major component of atherosclerotic lesions, foam cells play a particularly important role in the development of atherosclerosis. The formation of foam cells can promote the development of atherosclerosis (Mannarino and Pirro, 2008; Libby et al., 2011; Moore et al., 2013). Therefore, the reduction in the conversion of macrophages to foam cells will be an effective therapeutic strategy for reversing plaque lipid accumulation.

Cholesteryl esters (CEs), which are taken up into macrophage lipoproteins, are hydrolysed to free cholesterol (FC) and fatty acids. It has been found that the ATP-binding cassette transporters ABCA1 and ABCG1 play an important role in the transfer of FCs to the extracellular surface of the cells. Knockout of ABCA1 and ABCG1 in macrophages promotes atherosclerosis in mice (Westerterp et al., 2014).

Autophagy is a conserved cellular catabolism process in which the cytoplasmic components are encapsulated by autophagosomes that fuse with lysosomes to form autolysosomes, where they undergo substance degradation (Levine and Kroemer, 2019). The autophagy of cytoplasmic lipid droplets, also known as lipophagy, involves catalysis of the triglycerides stored in cells and promotes fatty acid oxidation to maintain cellular energy homeostasis (Singh et al., 2009). By increasing autophagy, cells can enhance the absorption and re-decomposition of oxidised LDL and acetylated lipoprotein, thereby enhancing plaque stability. In addition to the cholesterol transport pathway, autophagy-lysosome system is another important way to regulate intracellular cholesterol metabolism (Ouimet et al., 2011; Robinet et al., 2013).

Tongxinluo (TXL) is a traditional Chinese medicine made of 12 kinds of animal and plant products. Since its approval by the State Drug Administration of China in 1996, it has been widely used in the treatment of various cardiovascular diseases such as atherosclerosis (Chen et al., 2009; Hao et al., 2015). A large number of clinical and basic studies have found that TXL has anti-atherosclerotic effects, including improvement of the stability of atherosclerotic plaques, inhibition of systemic inflammation, and regulation of lipid metabolism. Using mouse genechip (Ma et al., 2019), it was found that 114 genes in the aortic tissue of atherosclerosis animal model were modified, including 48 genes that were up-regulated and 56 genes that were down-regulated in atherosclerosis. In the TXL treatment group, these changes were reversed. One of these genes is lectin-like oxidised low density lipoprotein receptor 1 (LOX-1), which is one of the scavenger receptors for oxidised LDL cholesterol (ox-LDL) and plays a vital role in the uptake of ox-LDL in cells (Pothineni et al., 2017).

TXL can effectively delay the progression of atherosclerotic lesions (Zhang et al., 2019). A multicentre randomised double-blind parallel group placebo-controlled study found that TXL decreased the mean carotid intima-media thickness (IMT), plaque area, and the progress of vascular remodeling. And it also reduced the incidence of unstable angina. Anti-inflammation and regulation of lipid metabolism are important anti-atherosclerotic mechanisms of TXL (Zhang et al., 2018; Ma et al., 2019). However, the mechanism of TXL on autophagy-mediated lipid homeostasis is not clear.

The purpose of this study was to examine and describe the therapeutic effects of TXL on lipid metabolism in macrophages both *in vitro* and *in vivo*, and to explore the underlying mechanisms.

## Materials and Methods

### Ethics Statement

All experiments *in vitro* were approved by the Key Laboratory of Cardiovascular Remodelling and Function Research, Qilu Hosipital, China. All *in vivo* protocols involving animal care and experiments complied with the Guide for Care and Use of Laboratory Animals published by the National Institutes of Health, United States (8th Edition, 2011) and the Animal Management Rules of the Chinese Ministry of Health (Project No. 55, 2001).

### Preparation of Tongxinluo

TXL ultrafine powder was obtained from Yiling Pharmaceutical Co. Ltd. (Shijiazhuang, Hebei, China). For *in vitro* experiments, TXL ultrafine powder was dissolved in serum-free RPMI 1640 medium (Gibco, United States) with the ultrasound technology to melt it well. The solution was centrifuged at 3,500 rpm for 10 min, and the supernatant was filtered with Sterile Syringe Filters (Millex-GP Syringe Filter Unit, 0.22 µm, Merck KGaA, Darmstadt, Germany). For *in vivo* experiments, TXL ultrafine powder was dissolved in saline and was administrated to mice daily.

### Cell Culture

Human acute monocytic leukaemia cell line (THP-1) was obtained from American Type Culture Collection (ATCC) and was cultivated in RPMI 1640 medium supplemented with 10% foetal bovine serum (FBS) and 1% penicillin/streptomycin at 37°C in 5% CO2. For adherence and differentiation of THP-1 cells into macrophages, 160 nM phorbol myristate acetate (PMA) was used overnight. THP-1 macrophages were incubated with or without TXL for 24 h before being stimulated with 50 mg/L recombinant human ox-LDL for indicated time.

### siRNA and RNA Interference

Upon reaching 40–60% confluence, HUVECs were transfected with specific siRNA or negative control siRNA (GenePharma, Shanghai, China) using Lipofectamine 3,000 (Thermo Fisher Scientific, Waltham, MA, United States) in Opti-MEM (Gibco, Thermo Fisher Scientific, Waltham, MA, United States). After 6 h of transfection, the medium was replaced with complete 1,640, and the cells were cultured for an additional 24 h. The transfected cells were treated with ox-LDL at the designated concentrations and for the indicated times. The DNA target sequence for Beclin-1 siRNA is 5’-CAG​TTT​GGC​ACA​ATC​AAT​A-3’.

### Protocol for Development of Atherosclerosis Animal Models

Male apoE^−/-^ mice (8 weeks old, 18–23 g) were purchased from the Peking University Animal Research Center (Beijing). All mice were fed atherogenic chow (1.25% cholesterol and 40% cocoa butter). The atherosclerotic models were created as previously described (Zhang et al., 2014). The mice were randomly divided into four groups (*n* = 24 per group): normal saline group (NS), low-dose TXL group, which received an oral dose of 0.38 g/kg/day TXL, medium-dose TXL group, which received an oral dose of 0.75 g/kg/day TXL, and high-dose TXL group, which received an oral dose of 1.5 g/kg/day TXL. Four weeks after the carotid-artery surgery, a 200 μl of suspension (4*10^8^ TU Beclin-1i per ml) was injected into each mouse through the tail vein. All mice underwent euthanasia 4 weeks post-transfection.

### Lentiviral Silencing

The lentivirus vector pGLV3/H1/GFP + Puro (pGLV3) was purchased from GeneChem (Shanghai, China) and a short-hairpin RNA sequence targeting Beclin-1 and scrambled control RNA were cloned into the vector. The following duplexes targeted murine Beclin-1: sense 5’-UAA​UAU​UAA​ACC​ACA​UGU​UUA​CA-3’, antisense 5’-UGU​AAC​GGA​UCC​UUA​ACA​AAU​GU-3’.

### Measurements of Serum Biological Parameters

Blood samples were collected by retro-orbital blood. After incubation at room temperature for 30 min and serum was separated by centrifugation (4°C, 2,500 r.p.m., 20 min). Lipid groups including TC, total TG, LDL cholesterol and HDL cholesterol were detected by automatic chemical modification technology (Roche Modular DPP System, Roche, Basel, Switzerland).

### Immunofluorescence Staining and Microscopy

The aortic roots were dissected, fixed in 4% formaldehyde overnight at 4°C, embedded in OCT compound, and prepared into 5-μm-thick sections. The cryosections or cell slides were blocked with 5% BSA and were incubated with primary antibodies at 4°C overnight. The sections were washed with PBS and were incubated with Alexa Fluor 488 or Alexa Fluor 594 conjugated secondary antibodies. LDs were stained with BODIPY 493/503 (Thermo, D-3922). Autophagolysosomes were stained with LysoTracker Red (Beyotime, C1046). Nuclei were stained with 4’, 6-diamidino-2-phenylindole (DAPI, 1:2000, Roche, Mannheim, Germany) for 15 min. The samples were rinsed three times in PBS and were examined under an epifluorescence microscope (Nikon, Japan).

### Western Blot Analysis

THP-1 macrophages and tissue samples were lysed using Minute™ Protein Extraction Kits containing 1 mM phosphatase inhibitors and protease inhibitor (Invitrogen, Carlsbad, CA, United States) and were collected after centrifugation at 16,000 × rpm for 10 min. The protein concentrations were determined using a BCA assay kit. Equal amounts of proteins and pre-stained protein ladder (Thermo Fisher Scientific) were separated on 12% SDS-PAGE gels, transferred to the Immovilon PVDF membranes (Millipore, Billerica, MA, United States), and incubated with primary antibodies overnight at 4°C. The membranes were incubated with secondary antibodies (ProteinTech, Rosemont, Penn., United States) the next day for overnight at 4°C. Bands were visualised using Immobilon ECL substrate (Millipore, Billerica, MA, United States), and blots were imaged with an Amersham Imager 600 (GE, United States). Protein expression was quantified using Adobe Photoshop CS6 (Adobe Systems, San Jose, CA, United States), normalised to the GAPDH expression in each sample, and expressed as percentage of the control.

### Reagents

Antibodies used for immunoblotting were as follows: LC3B antibody (abcam 51,520), ABCA1 antibody (abcam 7,360), ABCG1 antibody (abcam 52,617), LOX-1 antibody (abcam 60,178), HDAC one antibody (abcam 109,411), HDAC two antibody (abcam 32,117), HDAC three antibody (abcam 32,369), HDAC eight antibody (abcam 187,139), GAPDH antibody (ptg 60,004–1), Beclin-1 antibody (CST #3495). 3-MA (Sigma-Aldrich, 5 mM). Trichostatin A (TSA) (MCE HY-15144, 300 nM) and ITSA-1 (MCE HY-100508, 50 μm).

### Statistical Analysis

Data were analysed using SPSS software v16.0 (SPSS Inc., Chicago, IL, United States). Data were presented as the mean ± S.E.M. of at least three independent experiments. The normality of variable distribution was tested by the Kolmogorov–Smirnov test. Comparisons were analysed using Student’s *t*-test or one-way ANOVA followed by Bonferroni post hoc test. *p* < 0.05 was considered statistically significant.

## Results

### Tongxinluo Treatment Inhibited Lipid Deposition in Advanced Atherosclerotic Plaques by Enhancing Macrophage Autophagy

**FIGURE 1:**
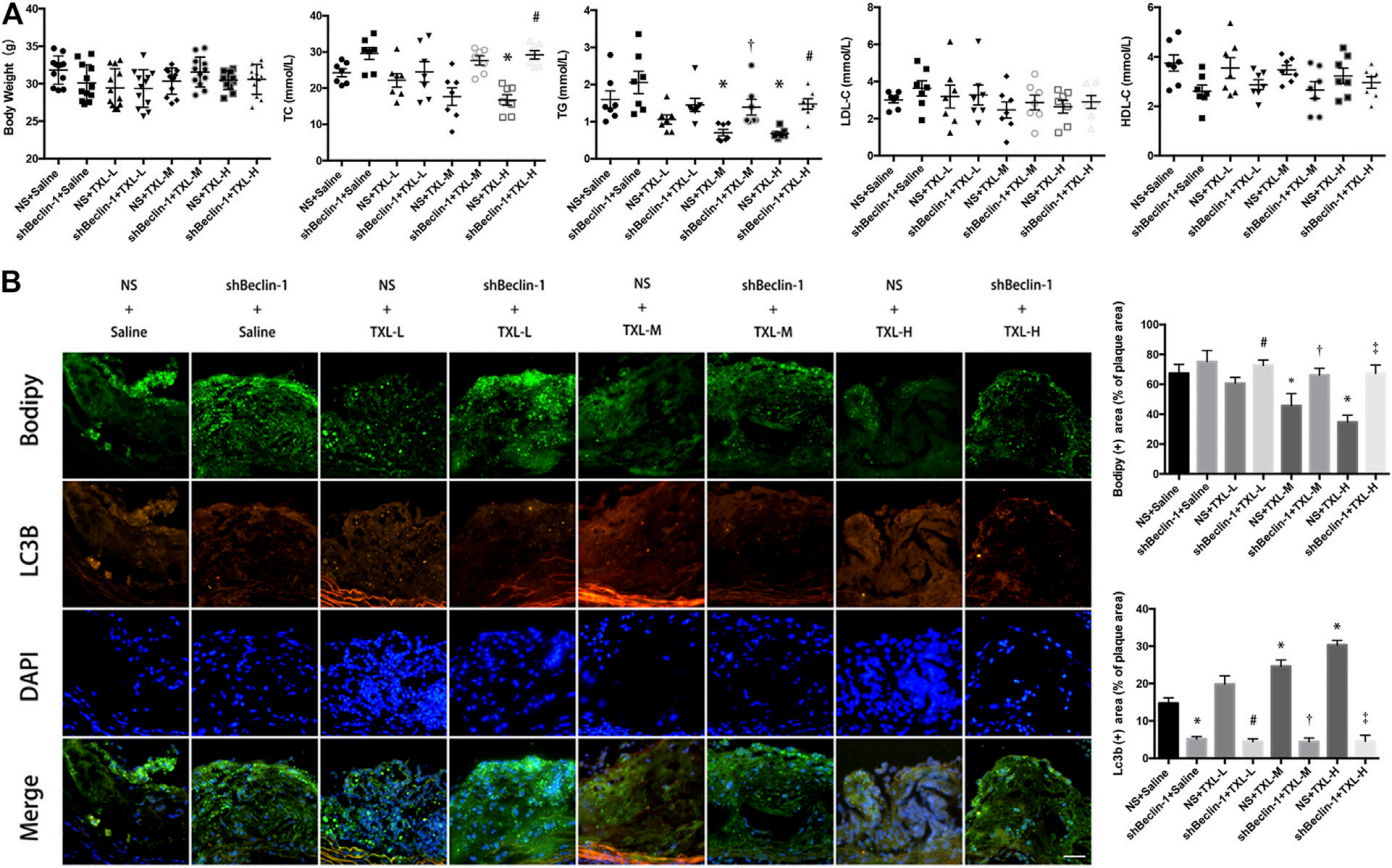
TXL treatment inhibited lipid deposition in advanced atherosclerotic plaques. **(A)** Mouse body weight and blood lipids, *n* = 7, *p < 0.05 vs. NS + Saline, ^#^ *p* < 0.05 vs. NS + TXL-H, ^†^ *p* < 0.05 vs. NS + TXL-M. **(B)** Effects of TXL on autophagy and lipid deposition in advanced atherosclerotic plaques, *n* = 3, bar = 50 μm *p < 0.05 vs. NS + Saline, #*p* < 0.05 vs. NS + TXL-L, ^†^ *p* < 0.05 vs. NS + TXL-M, ^‡^ *p* < 0.05 vs. NS + TXL-H.

We used low-, medium-, and high-dose TXL treatment (0.38, 0.75, and 1.5 g/kg/day, respectively) for apoE−/− mice. There was no difference in mouse body weight between experimental groups. The TC and TG values of NS + TXL-H group were lower than those of NS + Saline group (*p* < 0.05), and the TC and TG values of shBeclin-1 + TXL-H group were higher than those of NS + TXL-H group (*p* < 0.05), while there was no significant difference in the values of HDL-C and LDL-C between the groups (Figure 1A).

To test whether TXL reduces lipid accumulation in atherosclerotic plaques by promoting autophagy in macrophages, we used lentivirus to silence Beclin-1, which was injected in the tail of mice. Then autophagosomes were labelled with LC3B and lipids were labelled with BODIPY. Co-localised staining of lipid droplets and autophagosomes in mouse aortic root plaques was observed. In the NS + TXL-M and NS + TXL-H groups, the lipid content in the plaque decreased (*p* < 0.05, Figure 1B), and the level of autophagy was increased compared with the NS group (*p* < 0.05). However, this difference was not observed in the shBeclin-1 groups. In connection with the earlier research findings (Chen et al., 2018), these data indicate that TXL improves lipid deposition in advanced atherosclerotic plaques, and this effect is induced by autophagy.

### Tongxinluo Treatment Inhibited Lipid Deposition in THP-1 Macrophages and the Formation of Foam Cells

**FIGURE 2:**
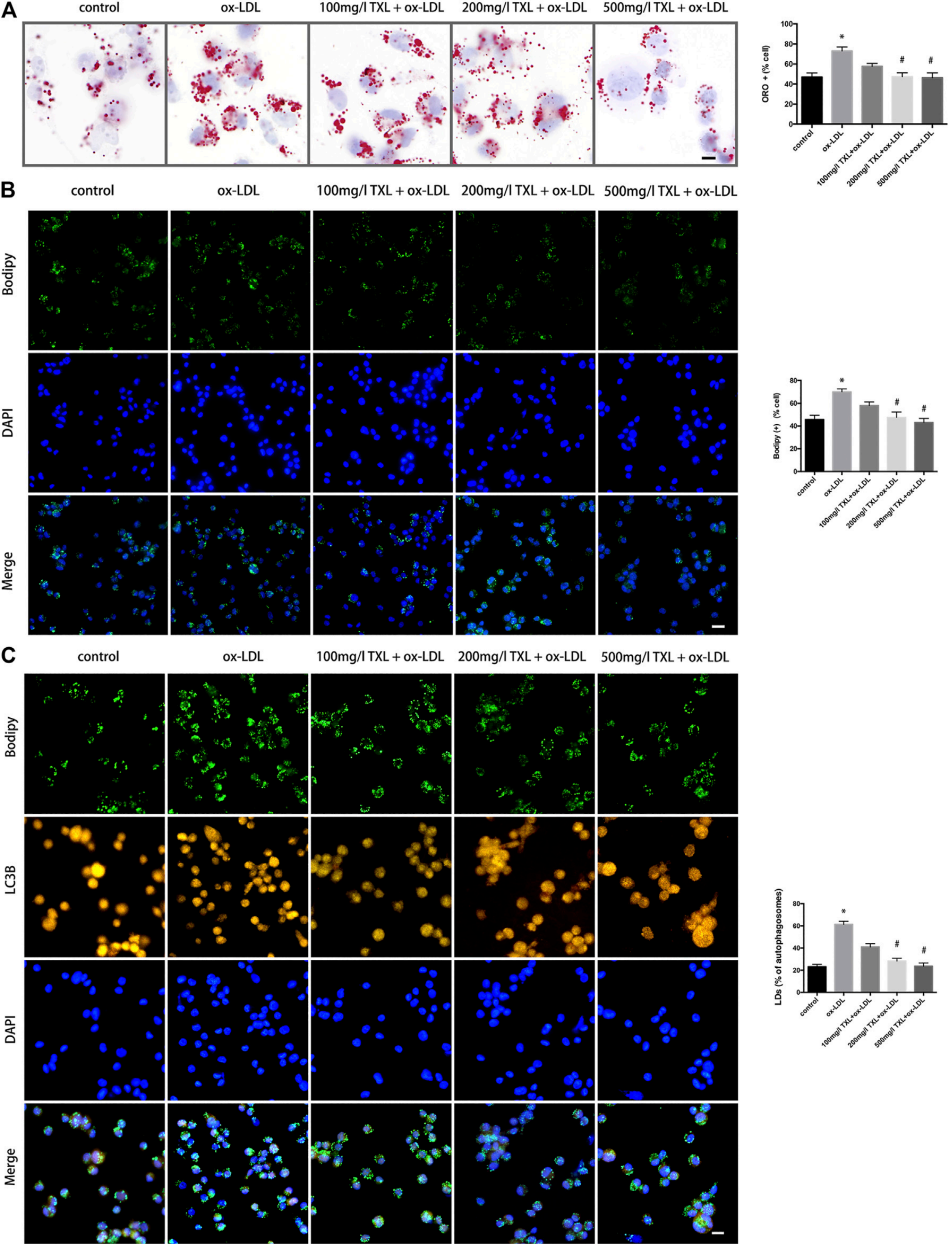
TXL treatment inhibited lipid deposition in THP-1 Macrophages. **(A)** Cell oil red O detected the effect of TXL on ox-LDL-induced macrophage lipid deposition, *n* = 3, bar = 10 μm, *p < 0.05 vs. control, ^#^ *p* < 0.05 vs. ox-LDL. **(B)** Cell Bodipy staining detected the effect of TXL on ox-LDL-induced macrophage lipid deposition, *n* = 3, bar = 25 μm, *p < 0.05 vs. control, ^#^ *p* < 0.05 vs. ox-LDL. **(C)** Effects of TXL on autophagy and intracellular lipid in macrophages. *n* = 3, bar = 10 μm *p < 0.05 vs. control, ^#^ *p* < 0.05 vs. ox-LDL.

To study the effect of TXL on the formation of ox-LDL-induced macrophage foam cells, the successfully induced macrophages were pre-treated with TXL, stimulated with ox-LDL, and detected by oil red O staining and BODIPY staining. As shown in Figures 2A,B, ox-LDL caused an increase in lipid content in macrophages compared to the control group (*p* < 0.05). Compared with the ox-LDL group, the lipid content in the macrophages of 200 mg/L TXL + ox-LDL and 500 mg/L TX + ox-LDL was reduced (*p* < 0.05), and the inhibitory effect of 500 mg/L TXL was more pronounced. Green fluorescence is expressed as a positive concentration of LDs in the macrophage, and green fluorescence in the ox-LDL group is stronger than that in the control group (*p* < 0.05), indicating that ox-LDL induces lipid deposition in macrophages to foam cells. After treatment with TXL, the green fluorescence in macrophages decreased in a dose-dependent manner (*p* < 0.05), indicating that TXL can inhibit ox-LDL-induced macrophage lipid deposition.

LDs is where the foam cells store cholesterol. Reducing the foaming and lipid deposition of macrophages is a potential therapeutic target for reversing atherosclerosis. In order to explore the effect of TXL on the breakdown of lipids LDs, autophagosomes were labeled with LC3B antibody and LDs were labelled with BODIPY. Compared with the ox-LDL group, in 200 mg/L TXL + ox-LDL and 500 mg/L TXL + ox-LDL groups, the number of lipid LDs co-localised with autophagosomes was decreased (*p* < 0.05, Figure 2C).

### Tongxinluo Promotes Lipid Degradation in Lipid Droplets by Enhancing Autophagy

**FIGURE 3:**
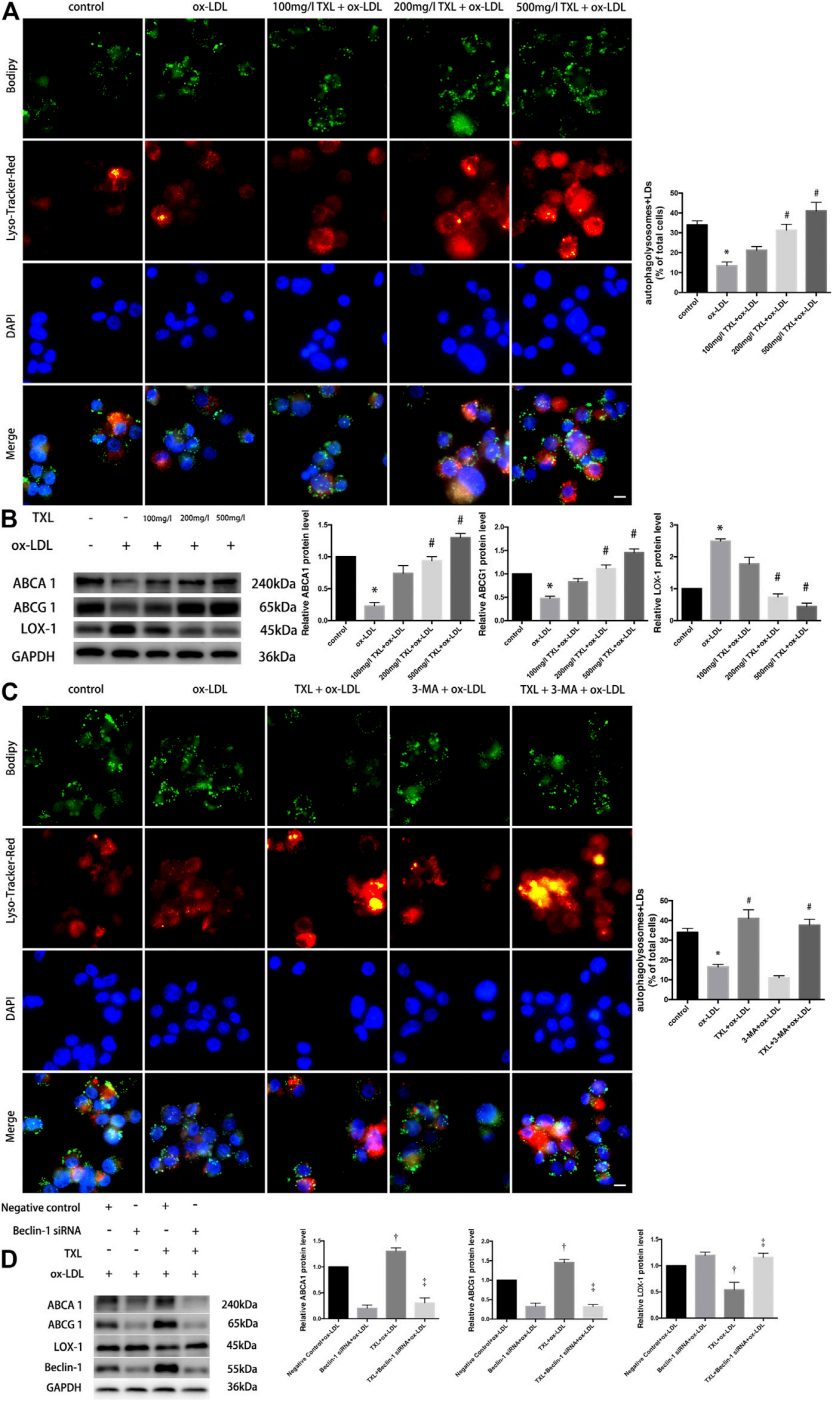
TXL treatment reduced lipid deposition by increasing autophagy in THP-1 Macrophages. **(A)** Effects of TXL on intracellular lipid metabolism of macrophages mediated by autophagy, *n* = 3, bar = 10 μm, *p < 0.05 vs. control, ^#^ *p* < 0.05 vs. ox-LDL. **(B)** Western Blot detection of TXL on ox-LDL-induced macrophage lipid efflux, *n* = 3, *p < 0.05 vs. control, ^#^ *p* < 0.05 vs. ox-LDL. **(C)** Detection of autophagy in TXL inhibited ox-LDL-induced macrophage lipid deposition *via* cellular immunofluorescence, *n* = 3, bar = 10 μm, *p < 0.05 vs. control, ^#^ *p* < 0.05 vs. ox-LDL. **(D)** Western blot revealed the effect of Beclin-1 in inhibition of ox-LDL-induced lipid deposition in TXL-treated macrophages. *n* = 5, ^†^ *p* < 0.05 vs. Negative control + ox-LDL, ^‡^ *p* < 0.05 vs. TXL + ox-LDL.

To explore the effect of TXL on the autophagic outflow of lipid droplets in macrophages, autophagy lysosomes were labeled with LysoTracker Red and LDs were labelled with BODIPY. Co-localisation of BODIPY and LysoTracker Red showed that in 200 mg/L TXL + ox-LDL and 500 mg/L TXL + ox-LDL groups, the number of lipid LDs co-localised with autophagosomes was increased, more lipids were co-localised with autophagy lysosomes (*p* < 0.05, Figure 3A). And in Figure 3B, ox-LDL stimulation by western blot decreased the expression of ABCA1, ABCG1 protein expression and increased the expression of LOX-1 protein expression, but pretreatment of TXL significantly inhibit it with the effects at 200 mg/L and 500 mg/L concentrations (*p* < 0.05, Figure 3B).

After macrophages were pre-treated with TXL and ox-LDL stimulation, macrophage autophagy was quantified by co-localisation with BODIPY and LysoTracker Red, with or without autophagy inhibitor 3-MA. As shown in Figure 3C, the number of autophagolysosomes in the macrophages of the 3-MA + ox-LDL group was lower than that of the control group. After treatment with TXL, more lipids in the macrophages of the ox-LDL group were bound to autophagosomes (*p* < 0.05), and the number of autophagosomes became higher. Lipid deposition in 3-MA + ox-LDL group was significantly higher than that in TXL + ox-LDL group, but it was lower in 3-MA + TXL + ox-LDL group and in the TXL group. The autophagic level of the lipid droplets in the cells was increased (*p* < 0.05). These results suggested that TXL reduced lipid deposition by increasing autophagy levels.

To show the role of autophagy-related gene Beclin-1 in inhibition of ox-LDL-induced lipid deposition in TXL-treated macrophages, Beclin-1 gene was silenced using siRNA. Compared with the TXL + ox-LDL group, ABCA1, ABCG1 protein expression decreased and LOX-1 protein expression increased (*p* < 0.05) in TXL + Beclin-1 siRNA + ox-LDL group (*p* < 0.05, Figure 3D).

### Tongxinluo Inhibits the Expression of Histone Deacetylase to Increase Macrophage Autophagy

**FIGURE 4:**
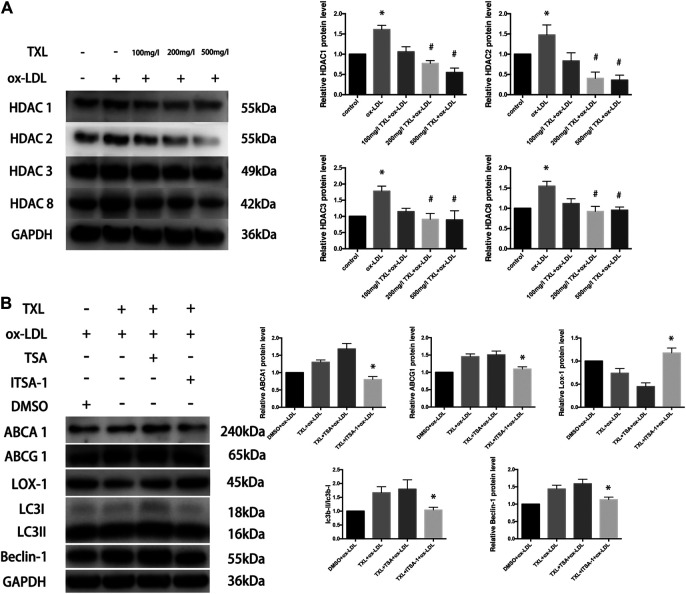
TXL increase macrophage autophagy through histone deacetylase inhibition. **(A)** Western blot revealed the effect of TXL on ox-LDL-induced macrophage type I histone deacetylase expression, *n* = 5, *p < 0.05 vs. control, ^#^ *p* < 0.05 vs. ox-LDL. **(B)** Western blot revealed histone deacetylase inhibited the effect of TXL on macrophage lipid deposition, *n* = 5, *p < 0.05 vs. TXL + ox-LDL.

In the past few decades, drugs targeting histone deacetylase (HDACs) have been identified and developed for the treatment of cancer, and more non-oncological applications of these drugs have been discovered. It has been confirmed that histone deacetylase can directly participate in the regulation of autophagy key proteins and autophagy-related proteins, to regulate autophagy. To explore the mechanism by which TXL regulates autophagy in macrophages, we suppose that TXL can alter HDAC regulate autophagy and observe the effect of TXL on HDAC. Compared with the control group, ox-LDL caused an increase in macrophage class I histone deacetylases (HDAC1, HDAC2, HDAC3, and HDAC8) protein expression (*p* < 0.05) while TXL reversed the HDACs high expression (*p* < 0.05) induced by ox-LDL (Figure 4A). In addition, compared with TXL + ox-LDL group, after addition of HDAC agonist (ITSA-1), the ratio of LC3II/LC3I, Beclin-1 protein expression, ABCA1 and ABCG1 protein expression decreased, and LOX-1 protein expression increased (*p* < 0.05, Figure 4B).

## Discussion

In this study, we investigated how TXL affects lipid deposition in advanced atherosclerotic plaques and lipid metabolism in macrophages. The following conclusions were generated: 1) TXL treatment inhibited lipid deposition in advanced atherosclerotic plaques by enhancing macrophage autophagy. 2) TXL treatment inhibited lipid deposition in THP-1 macrophages and the formation of foam cells. 3) TXL promotes lipid degradation in lipid droplets by enhancing autophagy. 4) TXL inhibits the expression of histone deacetylase to increase macrophage autophagy.

To evaluate the role of TXL in atherosclerotic plaques, we construct the atherosclerotic animal model with lentiviral injection and performed histopathology and immunofluorescence staining analysis *in vivo*. Immunofluorescence staining revealed that TXL treatment inhibited lipid deposition in advanced atherosclerotic plaques by enhancing macrophage autophagy. *In vitro* TXL treatment inhibited lipid deposition in THP-1 macrophages by enhancing autophagy *via* Beclin-1. TXL reversed the high expression of class I histone deacetylases (HDACs) induced by ox-LDL. Trichostatin A (TSA) is a specific inhibitor of class I histone deacetylase. ITSA-1 is an agonist of histone deacetylase. In order to study the effect of class I histone deacetylase on TXL treatment, we added TSA or ITSA-1 to THP-1 macrophages. Compared with the TXL + ox-LDL group, TXL failed to promote intracellular lipid droplet decomposition after the addition of the histone deacetylase agonist.

Atherosclerosis is a progressive disease characterised by the accumulation of lipid and fibre elements in the aortic walls, leading to myocardial infarction or stroke. Tongxinluo (TXL), a traditional Chinese medication, plays a key role in the formation and progression of plaques in atherosclerosis. In previous research (Chen et al., 2018), we had found that TXL significantly inhibits ox-LDL-induced apoptosis in macrophages *in vitro* by improving the dissociation of the Beclin-1-Bcl-2 complex. *In vivo*, TXL treatment significantly reduced macrophage apoptosis dose-dependently and the result was blocked by Beclin-1 silencing. In addition, the increased Lc3b dots by TXL almost localized to macrophages in advanced atherosclerotic plaque. Compared with the same dose of TXL shBeclin-1 group, plaque area (showed by positive oil red O-stained area of en face staining or staining of aortic root sections with H&E) and the vulnerability index of TXL groups decreased. The anti-apoptosis effects of TXL on atherosclerosis was related to the improvement of autophagy *via* Beclin-1. Our current study reveals that TXL inhibits the lipid deposition through increasing autophagy related Beclin-1 both *in vitro* and *in vivo*. TXL also reduces the expression of histone deacetylase, which are involved in autophagy inhibition and counteracts atherogenic effect of ox-LDL in THP-1 macrophages.

After apolipoprotein E (apoE) knockout animals were exposed to a high-fat diet, lipoprotein particles and their aggregates accumulated in the damaged intima, and mononuclear cells adhered to the surface of the endothelium migrate through the endothelial monolayer to the intima. Then, the cells proliferate and differentiate into macrophages and ingest lipoproteins, forming foam cells. Over time, the foam cells undergo apoptosis, lysis, and their lipid-filled contents turn into the necrotic core of the plaque (Ross, 1993; Tamminen et al., 1999). The accumulation of ox-LDL in the intima significantly promotes monocyte recruitment and foam cell formation (Cyrus et al., 1999; Libby, 2000; Maxfield and Van Meer, 2010). In the present research, *in vivo* experiments showed that the accumulation of lipids in macrophage promotes the development of atherosclerotic plaques, so TXL maybe inhibit the progress of atherosclerotic plaques by attenuating the accumulation of lipid in macrophage by enhancing Beclin-1-induced autophagy. *In vitro* experiments showed that TXL could inhibit macrophage lipid deposition induced by ox-LDL. However, after the Beclin-1 gene was silenced, these effects disappeared, indicating that TXL achieves its effects through autophagic key molecule Beclin-1.

The cell itself has a complete and complex mechanism to ensure the distribution and content of free cholesterol in order to prevent the cell membrane from being destroyed due to excess of cholesterol (Maxfield and Van Meer, 2010). In the state of increased cholesterol levels, cellular feedback mechanisms can reduce cholesterol uptake and synthesis and increase reverse cholesterol transport (Maxfield and Tabas, 2005). If these compensatory mechanisms do not adequately reduce free cholesterol levels, excess free cholesterol will be esterified and stored as lipid-free non-cytotoxic cholesterol esters to maintain normal cell function. The export of cholesterol mediated by autophagy is an ABCA1-dependent process (Orsó et al., 2000; Neufeld et al., 2001). ABCA1 promotes intracellular cholesterol efflux and increases high-density lipoprotein levels to achieve reverse cholesterol transport (RCT). Through RCT, i.e., cholesterol is transferred from macrophages, cleared by the liver, and eventually excreted (Chen et al., 2001; Cuchel and Rader, 2006). This study confirmed that TXL can up-regulate the expression of ABCA1 and ABCG1 protein and inhibit the expression of LOX-1 protein. This indicates that TXL can promote the RCT by initiating Beclin-1 mediated autophagic pathway, in order to achieve the inhibition of atherosclerotic plaque lipid deposition and to improve plaque stability.

LDs are metabolically active but atypical intracellular organelles composed of a hydrophobic core of neutral lipids (Ouimet and Marcel, 2012). In eukaryotic cells, various forms of autophagy can maintain the homeostasis of the intracellular environment and thus regulate cell survival. Experimental studies have found that autophagy dysfunction is associated with cancer, neurodegenerative diseases, inflammatory diseases, and immunodeficiency diseases (Ouimet et al., 2011). Autophagy, by releasing excess cholesterol, prevents the harmful effects of excessive cellular cholesterol accumulation in macrophages. The main mechanism is that cholesterol esters are stored in LDs, soluble in lysosomes, and hydrolysed by lysosomal acid lipase (LAL) into free cholesterol, then flows out of the cell (Sergin et al., 2017). Animal experiments indicated that in plaques of advanced atherosclerosis, autophagy occurs in macrophages. TXL promoted autophagy in plaque macrophages and suppressed lipid deposition in plaques. In the results of cell experiments, after TXL treatment, the more autophagic macrophages, the less lipid deposition, and most of the lipid was bound to autolysosomes, leading to significant increase in the volume of lysosomes. TXL also reversed the effect of inhibition of autophagy caused by 3-MA, but after silencing of Beclin-1, the drug of TXL was inhibited. These results suggest that TXL can exert its effect of inhibiting macrophage lipid deposition through Beclin-1 mediated autophagy pathway.

Class I HDAC shares homology with yeast Tpd3, including HDAC1, HDAC2, HDAC3 and HDAC8. Moreover, they are expressed in various tissues and organs. HDAC1, HDAC2 and HDAC3 play a regulation role in gene expression in the nucleus, and HDAC8 can deacetylate a variety of non-histone proteins in the nucleus and cytoplasm (Gammoh et al., 2012). Class I and class IIa HDACs inhibit the initiation of autophagy by altering the transcription or expression of an important autophagy-related protein (e.g., Beclin-1) or an extension (e.g., ATG7 or LC3B) during nucleation. In contrast, class IIb HDACs can promote autophagy maturation. These family members are involved in the process of promoting the transfer of autophagosomes to lysosomes and fusion with lysosomes. Class I HDAC specific inhibitors can reduce the inhibition of autophagy and thus promote the initiation of autophagy flow (Schipper et al., 2014). Results of this study indicate that TXL can inhibit the expression of type I HDAC, and after addition of HDAC agonists, the effect of TXL on promoting the expression of autophagy and lipid efflux protein is inhibited. The HDAC agonist used in this study is not a type I HDAC-specific agonist, therefore, the regulation of other types of HDAC by TXL needs further study.

In summary, TXL can inhibit the expression of type I HDAC, thereby inhibiting the inhibitory effect of histone deacetylase on the expression of autophagic key molecule Beclin-1 and promoting the autophagic outflow of intracellular lipids. This study provides experimental basis for the clinical application of TXL in regulating lipid metabolism and stabilising atherosclerotic plaques.

## Data Availability

The raw data supporting the conclusion of this article will be made available by the authors, without undue reservation.
